# Gas Poisoning

**Published:** 1890-07-26

**Authors:** 


### GAS POISONING.

Dr. Alois Pollak, of Prague, reports a case of poisoning by coal gas, which very nearly proved fatal. The patient, a short, stout man, was found quite unconscious shut up in a small workshop, the atmosphere of which smelt strongly of gas. His skin was pale and cold, the mucous surfaces also pale, with a tinge of violet. Heart's action weak, irregular, and fluttering; the pulse about sixty-six in the minute, but often scarcely perceptible. The respiration slow, shallow, stertorous; the cheeks distended in expiration; the tongue far retracted. He was moved to a large, airy room, and warmly covered. The treatment employed was vigorous friction of the extremities, with stimulation of the skin by momentary cold splashing, and artificial respiration, which was continued until the natural act was re-established. No reaction whatever was observed until after a quarter of an hour, when a little colour was seen in the cheeks and the parts submitted to friction. At the end of three-quarters of an hour some muscular tone was perceptible, and artificial respiration could be suspended for short intervals, though the failure of the natural efforts quickly called for its resumption. After an hour he was able to swallow some hot, strong, black coffee, and half an hour later he first opened his eyes, but knew no one. Respiration now proceeded regularly, and the artificial respiration was discontinued. He then slept for eight hours, when he awoke feeling weak, with headache, retching, and eructations, which smelt strongly of gas, but was unable to resume his work for two days. Dr. Pollak naturally thought it was a case of attempted suicide, but the man explained that having heard that coal gas was good for asthma, he had shut himself in the room with the gas turned on, and should do so again if his breath was as bad as it had been that day.

